# NGF/BDNF–Trk/p75NTR signaling in osteosarcoma immunity: biomarkers and therapeutic opportunities

**DOI:** 10.3389/fimmu.2025.1719012

**Published:** 2025-11-26

**Authors:** Lin An, Yunqiang Zhuang, Heyang Sun

**Affiliations:** 1 Trauma Orthopedic Center, Ningbo No.6 Hospital, Ningbo, China; 2 Ningbo Clinical Research Center for Orthopedics, Sports Medicine & Rehabilitation, Ningbo, China; 3 Department of Hand Microsurgery and Plastic Reconstructive Surgery, Ningbo No.6 Hospital, Ningbo, China

**Keywords:** osteosarcoma, neurotrophins, nerve growth factor, tumor−associated macrophages, myeloid−derived suppressor cells, nerve–tumor crosstalk

## Abstract

Osteosarcoma is a primary bone malignancy in which outcomes for patients with metastatic or relapsed disease remain unsatisfactory despite optimized surgery–chemotherapy backbones. Recently, advances in cancer neuroscience have highlighted neurotrophins—nerve growth factor (NGF) and brain−derived neurotrophic factor (BDNF)—and their Trk/p75^NTR^ receptors as modulators of tumor behavior and immune tone, offering a new strategy to recondition the osteosarcoma microenvironment. Evidence has been accumulated with the real−world application of TRK inhibitors in fusion−positive cancers and anti−NGF biologics in bone pain, together with osteosarcoma specimens and functional models showing that NGF/BDNF signaling promotes invasion, angiogenesis, and immunosuppressive niches, while neuromodulatory agents may counter these programs. Notably, deployment of these agents as immunity enablers in an immunologically “cold” sarcoma remains controversial, with uncertainties around biomarker selection, pharmacodynamic monitoring, and rational combinations with checkpoint or cellular therapies. In this review, we summarized NGF/BDNF expression and receptor activity in osteosarcoma and conducted a translational, efficacy− and safety−oriented appraisal of tissue/biofluid biomarker readouts and drugging opportunities targeting neurotrophin and co−regulatory neural circuits. In addition, we further discussed the potential mechanisms by which neurotrophin and adrenergic pathways regulate the balance between microenvironmental immunosuppression and lasting anti−tumor immunity. The purpose of this article is to define NGF/BDNF as shapers of the osteosarcoma immune microenvironment and to delineate biomarker−guided therapeutic opportunities for clinical testing.

## 1 Introduction

Osteosarcoma is a genomically unstable primary bone malignancy in which outcomes for patients with metastatic or relapsed disease have remained unsatisfactory despite optimized surgery–chemotherapy backbones (1–3). Multiple immunogenomic datasets converge on a low baseline T-cell infiltrate and paucity of actionable neoantigens (4–6), and emerging data suggest that histologic subtype, differentiation state, and canonical genotypes (e.g., TP53/RB1 loss; NTRK alterations) can tune innervation patterns and receptor programs that shape neuro-immune signaling in bone—motivating biomarker-guided remodeling of the microenvironment to restore antitumor immunity.

The osteosarcoma immune microenvironment includes dendritic cells, cytotoxic and regulatory T cells, NK cells, and key myeloid targets—tumor-associated macrophages (TAMs), monocytic M-MDSC and granulocytic PMN-MDSC—as well as osteoclast precursors that shape bone–immune crosstalk (7–9). These constituents, together with soluble mediators (e.g., IDO, IL−10, TGF−β, VEGF) and stromal elements, enforce immunosuppressive programs that constrain effector priming and infiltration (10–12). Such organization yields “cold−to−warm” phenotypic heterogeneity across patients and lesions and provides rational entry points for combinatorial immunotherapy.

Against this backdrop, neurotrophin biology—too rarely foregrounded—emerges as a regulatory tier with immediate bearing on immune tone in bone malignancies. NGF and BDNF transmit signals through the high−affinity Trk receptors—TrkA (NTRK1) and TrkB (NTRK2), respectively—and through the low−affinity p75 neurotrophin receptor (p75^NTR/NGFR^) (13–15). Present within, and responsive to, the tumor microenvironment are the ligands, their receptors, and the peripheral nerves that elaborate them; via these neural inputs, vasculature, stromal programs, and immune cell state are modulated, while reciprocal tumor−derived cues foster neo−innervation (16–18). Converging cancer-neuroscience data indicate that peripheral circuits also engage the cholinergic anti-inflammatory pathway in a stepwise manner—α7nAChR (ligand-gated) activation → Ca²^+^ influx → JAK2–STAT3 coupling → NF-κB restraint—reducing IL-6/TNF while largely preserving phagocytosis and antigen presentation.

Equally pertinent, neurotrophins act directly on immune cells. Human monocytes/macrophages constitutively synthesize NGF and, under inflammatory stimulation, further increase its production; through NGF signaling, adhesion−molecule programs are regulated and phenotype transitions that dictate antigen handling and cytokine release are favored (19–24). Distinct polarization states are driven by mature NGF versus pro−NGF, underscoring the contextual nature of neurotrophin effects on myeloid biology. Expression of BDNF and NGF has also been documented across additional immune subsets—including T−cell clones (25, 26)—supporting a circuit in which neurotrophins function simultaneously as products of, and modulators within, immune responses.

Focused osteosarcoma studies now place NGF/Trk signaling as a driver of malignant progression with microenvironmental consequences (27, 28). In clinical specimens and functional models, NGF levels are elevated, correlate with stage, and promote motility and invasion via MMP−2 and MEK/ERK pathway engagement; notably, pharmacologic Trk inhibition reverses these behaviors *in vitro* and limits pulmonary metastasis *in vivo* (29–31). Taken together, these findings suggest that neurotrophin blockade can concurrently temper tumor−intrinsic aggressiveness and interrupt paracrine loops that shape myeloid activation and immune trafficking within bone lesions.

Within cortical bone, neural input includes sympathetic/sensory fibers plus cholinergic signaling from scarce parasympathetic fibers and non-neuronal ACh produced by osteoblasts/osteocytes (choline-acetyltransferase/VAChT); this ACh axis modulates OPG/RANKL, osteoclastogenesis, and chemokine cues that recruit myeloid cells (32–34). Consistent with this premise, the broader cancer-neuroscience literature argues that therapeutically targeting neural–tumor crosstalk can recondition antitumor immunity; however, denervation and aberrant neo-innervation may alter circuit access and thus the efficacy of neural interventions, warranting stratification by nerve density and phenotype.

In this review, we synthesize evidence for NGF/BDNF signaling as a regulatory pathway in osteosarcoma immunity; delineate mechanistic interactions linking neurotrophin receptors to tumor and immune compartments; define measurable biomarker readouts across tumor tissue and biofluids; and evaluate therapeutically actionable strategies that target this axis, including rational combinations with immunotherapy.

## 2 The status and mechanisms of neurotrophin signaling (NGF/BDNF; TrkA/TrkB/p75NTR) in osteosarcoma and its immune landscape

Across osteosarcoma lesions, neurotrophin ligands and receptors are detectable in tumor and stromal compartments that collectively determine metastatic fitness and immune tone. Clinical tissue analyses and functional models indicate that NGF is upregulated in osteosarcoma and enhances migratory and invasive programs through TrkA–MEK/ERK–MMP−2 signaling; pharmacologic interruption of Trk or MEK/ERK signaling reverses these phenotypes and limits metastatic traits in preclinical systems. These data place NGF–TrkA at the intersection of tumor−intrinsic motility and microenvironmental remodeling (35–37). The key nodes of this axis are shown in **Table 1** to clarify receptor–effector mapping that is necessary for interpreting tissue readouts and therapeutic hypotheses. In bone sarcoma contexts, BDNF–TrkB signaling promotes angiogenic programs via PLCγ/PKCα and HIF−1α−dependent induction of VEGF, a change expected to alter leukocyte trafficking and the nutrient landscape within tumors; although these findings were established in human chondrosarcoma, they delineate a TrkB−centered pathway that is operative in skeletal tumors and relevant to osteosarcoma vascular phenotypes (38, 39). Moreover, osteosarcoma cells may express cholinergic components (e.g., α7 nicotinic receptors/CHRNA7 and choline-acetyltransferase), implying that α7nAChR-directed co-therapies could, context-dependently, affect proliferation and migration, warranting receptor profiling before combination testing.

**Table 1:** Mechanistic map of NGF/BDNF signaling nodes relevant to osteosarcoma immunity.

Neurotrophin axis	Principal receptors	Core downstream signals	Direct tumor-cell programs	Expected microenvironmental consequences	Exemplar measurable readouts
NGF → TrkA	TrkA (high-affinity), p75^NTR^ (context co-receptor)	MEK/ERK; PI3K/AKT; PLCγ/PKC	MMP-2 induction; migration/invasion; survival under stress	Endothelial activation; increased adhesion molecule expression; macrophage activation toward pro-inflammatory states	IHC/IF for NGF and phospho-TrkA; ERK phosphorylation; MMP-2 protein/activity; ICAM-1/VCAM-1 on vasculature
BDNF → TrkB	TrkB (high-affinity), p75^NTR^ (context co-receptor)	PLCγ/PKCα → HIF-1α; MEK/ERK; PI3K/AKT	Proliferation; EMT-like transcriptional shifts; survival	VEGF-driven angiogenesis; altered vessel permeability affecting immune cell trafficking	IHC/IF for BDNF and phospho-TrkB; VEGF and HIF-1α levels; microvessel density
Ligand (mature NGF) vs proNGF	TrkA-dominant vs p75^NTR^-dominant engagement	Ca^2^+ signaling; JNK/caspase (with p75^NTR^ bias)	Differential motility and cytoskeletal states; apoptosis sensitivity	Divergent macrophage polarization and chemotaxis; changes in phagocytosis and secretome	Ratio of mature NGF:proNGF (ELISA); p75^NTR^ expression; macrophage phenotypic markers (CD68/CD163/CD206)
Neuro-immune interface	TrkA/TrkB and p75^NTR^ on immune cells	MEK/ERK; AP-1; NF-κB	–	Monocyte adhesion; macrophage polarization; T-cell retention/function within tumor	Flow cytometry/IHC for TrkA/TrkB/p75^NTR^ on CD45^+^ cells; ICAM-1 on endothelium; cytokine/chemokine panels

Neurotrophins also regulate immune compartments that dominate osteosarcoma biology. Human monocytes/macrophages constitutively produce NGF and increase its synthesis after inflammatory stimulation, establishing an autocrine and paracrine source of ligand within lesions. In inflamed synovia, NGF acting via TrkA–MEK/ERK–AP−1 augments ICAM−1 expression and promotes M1 polarization, illustrating that NGF can gate adhesion programs and macrophage state through canonical kinase cascades; analogous circuits are expected to operate in tumor-associated macrophages exposed to NGF−rich milieus (40, 41). Mature NGF and proNGF impose distinct macrophage phenotypes through differential engagement of TrkA and p75^NTR^, with mature NGF enhancing phagocytosis and growth−factor secretion, and proNGF favoring migratory, podosome−rich states; such ligand−form dependence helps explain context−specific myeloid behavior in bone tumors. T−cell sources of NGF have been described, indicating that adaptive lymphocytes can both sense and supply neurotrophin signals that influence retention and function within the tumor bed (42–44). At the tissue scale, tumor–nerve crosstalk contributes additional neurotrophic input; studies across solid tumors document neural remodeling and nerve−derived cues that shape angiogenesis, stromal activation, and immune effector function (45–47), providing a framework for how neurotrophins and adrenergic/peptidergic signals converge on antitumor immunity.

Within metastatic niches, host−derived growth factors robustly activate ERK signaling and increase anti−apoptotic MCL1 in osteosarcoma cells, supporting survival during lung colonization; this situates neurotrophin−responsive MAPK signaling within a broader growth−factor ecosystem and underscores that intercepting ERK−driven programs can influence both tumor persistence and the composition of immune infiltrates that are conditioned by MAPK activity (48–50). These observations support a mechanistic model in which NGF/BDNF signaling through TrkA/TrkB and p75^NTR^ orchestrates: (1) tumor−intrinsic migration, invasion, and survival through MAPK/ERK, PI3K/AKT, and PLCγ/PKC modules; (2) vascular and stromal reprogramming that modifies leukocyte access and cytokine gradients; and (3) myeloid and lymphoid behavior via direct receptor engagement that biases adhesion, chemotaxis, and polarization states. This model yields practical implications for biomarker development and drugging strategies pursued later in the review: phospho−TrkA/TrkB and downstream effectors as tissue readouts; quantification of NGF/BDNF, VEGF, and MMP−2 in tissues and biofluids; nerve density and neurotrophin−responsive gene signatures as microenvironmental indices; and class−selective Trk inhibitors as tools to pharmacologically test causality in combination with immunomodulators.

## 3 Biomarker readouts for the neurotrophin–immune axis in osteosarcoma

**Figure 1:**
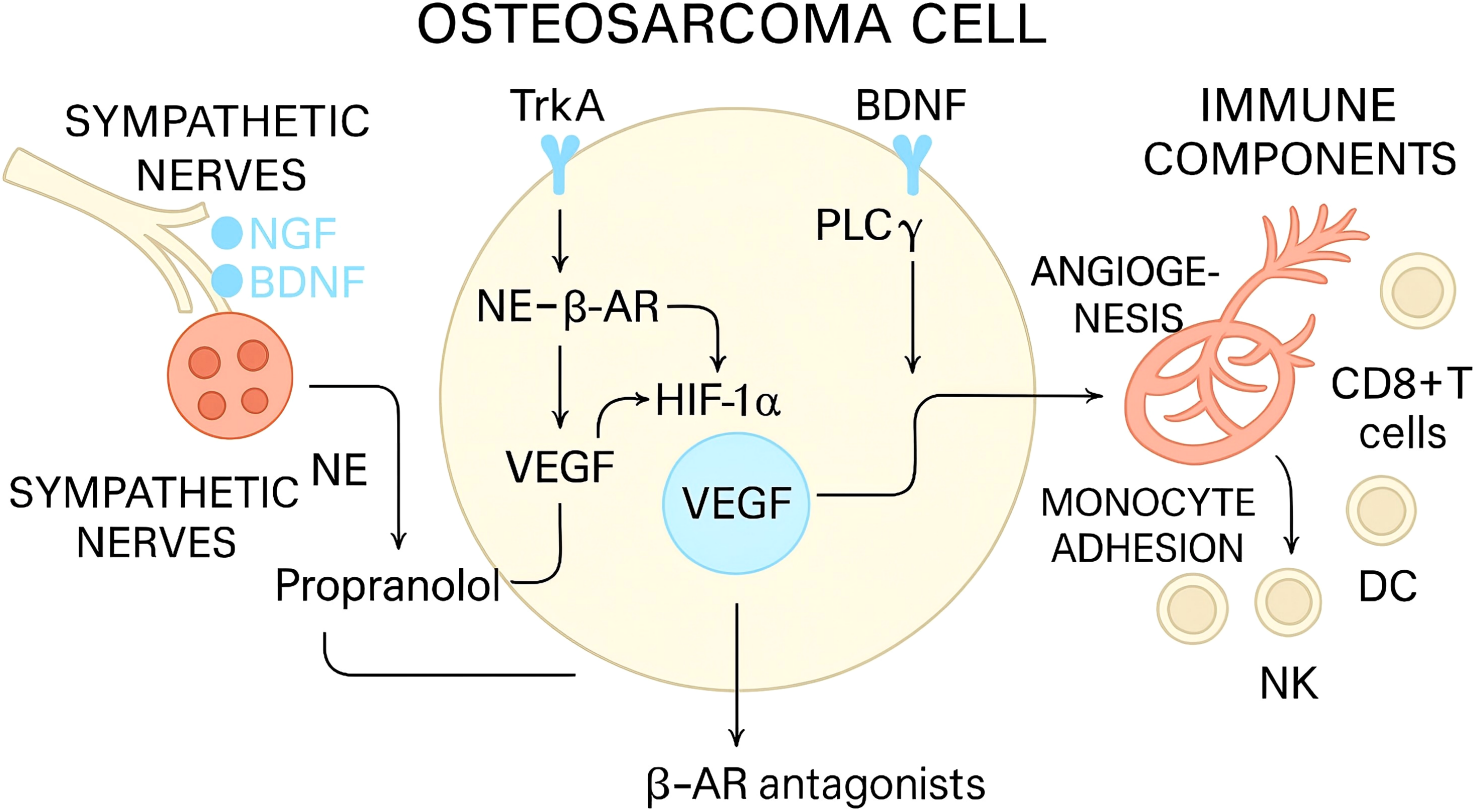
Sympathetic nerve–osteosarcoma crosstalk: Neurotrophin/β-Adrenergic Signaling Converges on HIF-1α–VEGF to Drive Angiogenesis and Immune Modulation.

Tissue−based readouts of neurotrophin pathway activity in osteosarcoma should prioritize direct ligand–receptor abundance and phosphorylation states together with downstream effectors and vascular correlates. Immunohistochemistry or multiplex immunofluorescence for NGF, BDNF, TrkA, TrkB, and phospho−Trk can be quantified by digital image analysis across tumor, stromal, and endothelial compartments. In osteosarcoma, NGF protein is elevated and correlates with stage, while NGF–MEK/ERK signaling drives MMP−2–linked motility; these observations justify inclusion of NGF, phospho−ERK, and MMP−2 as core readouts and support composite activity scores integrating receptor phosphorylation with protease expression (51–53). As shown in **Figure 1**, because BDNF–TrkB promotes VEGF via PLCγ/PKCα and HIF−1α in human chondrosarcoma, VEGF, HIF−1α, and microvessel density are rational proxies for TrkB−axis output relevant to osteosarcoma vasculature and immune trafficking (54–56). Pan−TRK immunohistochemistry is a pragmatic screening tool for TRK expression/fusions in routine pathology; however, mesenchymal tumors show context−dependent staining and physiologic expression that necessitate orthogonal confirmation by RNA/DNA assays and, for pathway activity, phospho−specific antibodies or phosphoproteomics (57–59). Host growth−factor–driven ERK phosphorylation and MCL1 upregulation support tumor cell survival in the lung, providing an additional downstream module to monitor in metastatic specimens and to correlate with immune infiltration patterns conditioned by MAPK signaling (60, 61). When feasible, spatial mapping of these markers relative to CD31^+^ endothelium, CD68^+^ myeloid cells, and CD8^+^ lymphocytes can refine interpretation of pathway–immune coupling within the tumor microenvironment.

Immune−facing readouts should capture neurotrophin receptor expression on leukocytes and adhesion programs that gate myeloid recruitment. Human monocytes/macrophages express TrkA and p75^NTR^ and exhibit ligand−form–dependent phenotypes; flow cytometry for TrkA/TrkB/p75^NTR^ on CD45^+^ subsets, coupled to ex vivo stimulation with mature NGF versus proNGF, can profile receptor competence and functional polarization states relevant to osteosarcoma lesions (62–64). Endothelial ICAM-1/VCAM-1 merit inclusion because NGF–TrkA–MEK/ERK–AP-1 increases these molecules, while ACh/α7nAChR signaling can restrain their expression and tune nitric-oxide–dependent vascular tone in bone, jointly shaping effector T-cell entry and response to ICB; analogous mechanisms are testable in osteosarcoma using quantitative immunostaining with CD31 (65, 66). Neuroanatomical indices complement molecular assays: nerve fiber density quantified by pan-neuronal markers (e.g., βIII-tubulin, PGP9.5 or pan-neurofilament) and lineage-specific markers (tyrosine hydroxylase for sympathetic and CGRP for peptidergic sensory fibers) in bone and tumor provide a structural surrogate for neurotrophin tone, and non-invasive autonomic indices—respiratory sinus arrhythmia/high-frequency HRV during cervical/auricular sessions—can verify vagal recruitment; prior human bone studies and NGF-dependent perivascular innervation models outline validated staining panels transferable to osteosarcoma (67, 68). Biofluid measurements (ELISAs for NGF/BDNF) are feasible but should be interpreted with attention to cellular sources, as circulating monocytes can contribute materially to BDNF/NGF pools; pairing serum assays with leukocyte phenotyping increases interpretability in translational studies (69–71). In humans, PMN-MDSC are CD11b^+^CD14^-^CD15^+^CD66b^+^ (± LOX-1) and M-MDSC are CD11b^+^CD14^+^HLA-DR^low/^-^CD15^-^; separating these guides PD endpoints in OS because therapies may differentially deplete or reprogram each subset and such shifts track effector T-cell entry.

## 4 Drugging opportunities targeting NGF/BDNF and co−regulatory circuits in osteosarcoma immunity

Multiple pharmacologic entry points exist to interrupt neurotrophin−dependent maintenance of an immunosuppressive osteosarcoma niche. Class−selective TRK inhibition is directly testable: in orthotopic osteosarcoma models, larotrectinib suppressed NGF−driven MEK/ERK–MMP−2 signaling and reduced lung metastasis, providing a tumor−intrinsic and microenvironment−relevant mechanism that can be paired with immune readouts (72, 73). Beyond fusion−positive disease, these data argue for using TRK blockade as a ligand−pathway inhibitor to decrease protease−mediated matrix remodeling and to normalize chemokine/adhesion gradients that shape leukocyte trafficking. In skeletal tumors, BDNF–TrkB activates PLCγ/PKCα→HIF−1α to induce VEGF and angiogenesis; interrupting this axis is predicted to improve vascular quality and T−cell access (74, 75). While entrectinib and larotrectinib have established activity across NTRK−fusion cancers, exposure–response analyses and integrated efficacy datasets underscore their acceptable benefit–risk profile for tumor−agnostic deployment, supporting careful exploration in osteosarcoma with neurotrophin−high states and planned correlative immunology (76–78). These therapeutic concepts and the associated biomarker are summarized in **Table 2**. Studies should pre-specify PD assays of dendritic-cell maturation (e.g., CD83), MHC-I/II and CD80/CD86 up-regulation under CAIP-activating regimens, with dosing/scheduling chosen to restrain NF-κB without driving sustained STAT3-dominant tolerogenic DC state.

**Table 2:** Therapeutic interventions targeting NGF/BDNF–Trk and neural co−regulatory pathways in osteosarcoma immunity: targets, mechanistic rationale, pharmacodynamic biomarkers, and combination strategies.

Intervention class	Representative agents	Primary target(s)	Mechanistic immune rationale in osteosarcoma	Anticipated pharmacodynamic biomarkers	Rational combination partners
TRK inhibitors (selective/pan)	Larotrectinib; Entrectinib	TrkA/TrkB	Decrease NGF/BDNF signaling → lower ERK activation, MMP-2 output, and VEGF-driven abnormal vasculature; enable effector T-cell entry	Phospho-Trk; phospho-ERK; MMP-2; VEGF; microvessel morphology; CD8^+^ density	PD-(L)1 blockade; MAPK inhibitors; cytotoxic chemotherapy
Anti-NGF biologics	Anti-NGF antibodies	NGF ligand	Dampen neurogenic inflammation and endothelial adhesion programs that impede leukocyte trafficking	NGF levels; ICAM-1/VCAM-1; cytokine panels; pain scores (as neurogenic surrogate)	TRK inhibitors; PD-(L)1 blockade; analgesic-sparing regimens
β-adrenergic blockade	Propranolol	β1/β2-AR	Reduce stress-induced pro-angiogenic and immunosuppressive signaling; potentially restore antigen presentation and cytotoxic function	Catecholamines; phospho-AKT; VEGF; endothelial/perivascular markers; T-cell activation indices	TRK inhibitors; chemotherapy; PD-(L)1 blockade
Receptor-biased neurotrophin modulators	TrkB/TrkC partial agonists/ligands	TrkB/TrkC (± p75^NTR^ bias)	Re-tune receptor signaling to limit pro-tumor outputs while preserving trophic signaling; test effects on myeloid polarization and T-cell retention	Phospho-TrkB/TrkC; HIF-1α; myeloid phenotype markers; chemokine gradients	TRK inhibitors; anti-angiogenic agents; adoptive cell therapy

Co-regulatory neural circuits—including cholinergic pathways that promote specialized pro-resolving mediators (e.g., resolvins, lipoxins)—offer complementary levers to reprogram myeloid states without broadly suppressing immunity. β−adrenergic signaling promotes pro−angiogenic and stress−response programs in sarcomas; propranolol reduced proliferation and angiogenesis and enhanced cisplatin efficacy in osteosarcoma xenografts, nominating β−blockade as a feasible adjunct to TRK inhibition and to immune checkpoint therapy where adrenergic tone blunts effector function (79, 80). Broader neurobiology reviews converge on adrenergic targets as druggable modifiers of tumor–immune interactions, supporting rational combinations with neurotrophin−pathway inhibitors to mitigate convergent MAPK and HIF−1α signaling and to favor antigen presentation and cytotoxic T−cell infiltration (81, 82). Anti-NGF biologics, evaluated in bone cancer pain models, reduce neurogenic inflammation and may alleviate chemokine/adhesion programs that restrict leukocyte entry; because nociception and opioid therapy can shift sympathetic–vagal tone and innate immune set-points, immunomonitoring is essential when layering onto standard therapy or TRK blockade (83, 84). Receptor−biased modulation may provide finer control of neurotrophin signaling: small−molecule TrkB/TrkC ligands from the neurodegeneration field demonstrate tractable pharmacology and pathway selectivity, motivating preclinical testing for microenvironmental and immune consequences in osteosarcoma.

Trial design should prioritize enrichment for neurotrophin-active tumors (NGF/BDNF expression, phospho-Trk, MMP-2/VEGF, nerve fiber density) and incorporate paired tissue–blood immunophenotyping (myeloid subsets, endothelial adhesion molecules, T-cell activation/exhaustion markers) before and after pharmacologic intervention, with sampling and stimulation timed to circadian variation in autonomic tone and cytokines. In combination frameworks, TRK inhibitors can be paired with PD−(L)1 blockade or adoptive cell therapies, with β−blockers or anti−NGF agents used to reduce neural−immune suppression and to improve vascular and cytokine tone.

## 5 Conclusion and future expectations

Converging mechanistic and translational observations support a model in which NGF/BDNF signaling shapes osteosarcoma biology at three interdependent levels: tumor cells, the vascular–stromal interface, and immune effectors. NGF acting through TrkA regulates endothelial adhesion programs and macrophage polarization via MEK/ERK–AP−1, while circulating and tissue−resident leukocytes are competent sources and targets of neurotrophins, establishing autocrine and paracrine loops within lesions (85–87). BDNF–TrkB can induce HIF−1α–dependent VEGF, providing a plausible axis through which neurotrophin tone remodels angiogenesis and leukocyte access in skeletal tumors (88–90). Clinically validated TRK inhibitors demonstrate durable, tumor−agnostic efficacy with acceptable safety in NTRK−fusion cancers, offering agents with which to pharmacologically interrogate neurotrophin−dependent circuits in osteosarcoma when accompanied by appropriate biomarker enrichment; pathology best−practice guidance underscores the need to complement pan−TRK immunohistochemistry with orthogonal molecular assays and, for pathway activity, phospho−specific readouts (91–93). Anti−NGF biologics, developed in pain medicine, have reproducible effects on cancer−related bone pain and neurogenic inflammation, suggesting a feasible avenue to modulate neurotrophin−linked microenvironmental states while simultaneously improving symptom control (94–96). These studies justify a translational agenda in which NGF/BDNF biology is positioned as an actionable determinant of immune tone in osteosarcoma.

Future work should prioritize prospectively designed, biomarker−driven trials that (1) enrich for neurotrophin−active tumors using multiplex immunohistochemistry for NGF/BDNF, TrkA/TrkB and phospho−Trk, digital quantification of endothelial ICAM−1/VCAM−1 and microvessel morphology, and spatial mapping of myeloid and T−cell compartments; (2) incorporate paired tissue–blood immunophenotyping to test whether TRK blockade or anti−NGF therapy reconditions macrophage states, adhesion programs, and effector T−cell infiltration; and (3) integrate CAIP-targeted strategies with chemotherapy, surgery, radiotherapy, and anti-resorptives (bisphosphonates/denosumab), anticipating effects on myeloid dynamics (e.g., MDSC contraction/redistribution) and on antigen release/exposure that can potentiate checkpoint responses. To ensure interpretability, studies should pre−specify pharmacodynamic thresholds for pathway inhibition (e.g., reductions in phospho−ERK or HIF−1α/VEGF outputs) and align clinical endpoints with immune remodeling metrics, including changes in CD8^+^ density and exhaustion markers. Given the rarity and heterogeneity of osteosarcoma, platform or basket trial architectures with harmonized correlative assays across centers will be essential for generating decision-quality evidence while avoiding underpowered subgroup analyses. When executed with these principles, testing of NGF/BDNF−directed agents alone and in combinations designed to normalize vascular and cytokine tone should determine whether neuromodulatory strategies can convert immunologically quiescent osteosarcoma into a treatment−responsive state.
